# Fertility in early breast cancer patients: integrating reproductive planning into oncology care

**DOI:** 10.3389/fendo.2025.1695667

**Published:** 2026-01-05

**Authors:** Antonella Turla, Anna Bellicini, Daniele Spada, Marcella Mandruzzato

**Affiliations:** 1 Oncology, ASST Valcamonica, Esine, Brescia, Italy; 2 Gynecology, ASST Valcamonica, Esine, Brescia, Italy

**Keywords:** fertility preservation, early breast cancer, pregnancy safety, reproductive counseling, cancer survivors

## Abstract

Fertility preservation (FP) and adequate counseling are crucial for young breast cancer patients in the early setting. We provide a brief summary of FP in young breast cancer patients, including current international guidelines, available FP treatments, and the safety of pregnancy. This review explores embryo/oocyte cryopreservation, ovarian tissue cryopreservation (OTC), and gonadotropin-releasing hormone agonists (GnRHa), along with evidence on temporary endocrine therapy interruption to attempt pregnancy. Despite strong recommendations, FP uptake remains low, underscoring the need for timely multidisciplinary counseling. Literature data underline that pregnancy can be considered after treatment, even for hormone receptor-positive and BRCA mutation-positive patients. Reproductive planning should be integrated into the oncologic treatment, balancing oncologic priorities with patients’ reproductive desires.

## 1 Introduction

The detection of early breast cancer in young women (BCY) is increasing worldwide, and some data suggest an upward trend in this specific population, despite the existence of some regional differences (1). Some long-term survivorship issues, such as fertility and reproductive health, are becoming more and more important in BCY, due to better outcomes resulting from improvements in screening, molecular subtyping, and systemic therapies. The risk of chemotherapy-related amenorrhea (CRA) is a well-known side effect of chemotherapy. Although CRA may be reversible some months or, less commonly, years after chemotherapy, most young patients do not recover ovarian function after one year of CRA (2). This condition translates into a significant source of stress, with major consequences for the mental and physical health of patients of childbearing age.

The importance of providing proper reproductive counseling is recommended by international guidelines, including those from the American Society of Clinical Oncology (ASCO) (3) and the European Society for Medical Oncology (ESMO) (4), which advise that such an assessment be carried out before starting systemic treatment, or as early as possible during the therapeutic process. Oncofertility counseling should be discussed even with patients who do not currently have an immediate desire for motherhood, in light of the potential future implications of such a choice. Nevertheless, some historical cohort studies show that FP services in young women often remain a medical unmet need, due to many factors. Letourneau et al. reported that only 5% of young patients affected by cancer, not only breast cancer, received proper counseling from fertility specialists, and 4% started a FP treatment before systemic oncologic therapy (5).

## 2 Review methodology

This review summarizes current clinical approaches to fertility preservation and reproductive planning in early breast cancer, intending to integrate FP into routine clinical practice to increase the quality of life of BCY. The literature review was conducted in accordance with the main international guidelines on FP, with particular attention to the studies cited within the guidelines themselves. A further literature review was conducted using the keywords “fertility preservation” and “breast cancer patients” on PubMed, selecting the most relevant studies from recent years (2015–2025).

## 3 Risk stratification and pregnancy safety

**Figure 1:**
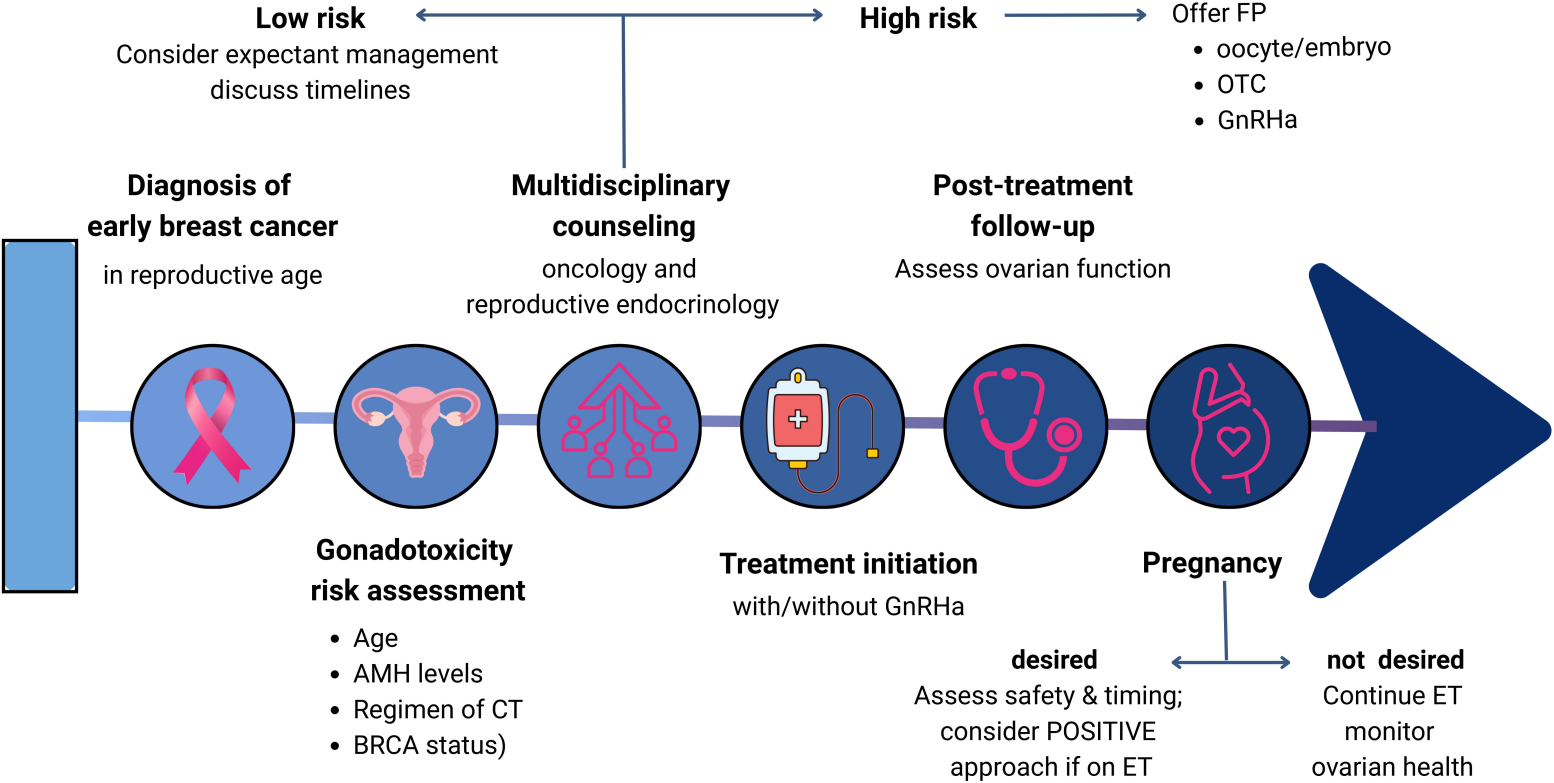
FP decision-making flowchart.

**Figure 1** summarizes the FP decision-making flowchart for pre-treatment and post-treatment reproductive planning, based on international guidelines and evidence in the literature.

Current ASCO (3) and ESMO (4) guidelines strongly recommend early counseling by a multidisciplinary team, ideally before systemic therapy begins. Assessment of gonadotoxic risk is the starting point for reproductive planning in early breast cancer. The evaluation of gonatoxicity risk has to consider many features, from patient age to baseline ovarian reserve, chemotherapy type, and cumulative dose.

Alkylating agents, such as cyclophosphamide, exert their gonadotoxic effects in two ways: they both induce amenorrhea related to the loss of the growing follicle population and reduce the primordial follicle pool (6). For this non-specific mechanism of action, alkylating agents are the most toxic drugs for FP. Doses greater than 4 g/m^2^ in males or >8g/m2 and 12 g/m^2^ in pubertal and prepubertal females, respectively, are considered high risk in cumulative exposure (7). Anthracycline- and taxane-based protocols also carry significant risk, though less than cyclophosphamide-based regimens. The addition of docetaxel at a dose of 100 mg/m² to the FEC regimen (5-fluorouracil 500 mg/m², epirubicin 100 mg/m², cyclophosphamide 500 mg/m²) appeared to harm ovarian reserve one year after diagnosis, compared to patients treated with FEC alone. However, no differences were reported in long-term outcomes (8).

The influence of anti-HER2 targeted therapies, such as trastuzumab and pertuzumab, on FP is less clear. Patients receiving paclitaxel associated with trastuzumab have low rates of treatment-related amenorrhea (9). Some data suggest no significant differences among drugs included in this category on treatment-related amenorrhea (10). They may indirectly delay conception due to treatment schedules.

Endocrine therapies (tamoxifen, aromatase inhibitors) can lead to temporary amenorrhea, usually not to an irreversible reduction of ovarian reserve (11). A study revealed no statistically significant alteration in serum anti-Müllerian hormone (AMH) levels over 24 months of tamoxifen treatment, after accounting for the age-related decline (12). The impact of GnRH analogues and aromatase inhibition on fertility remains to be thoroughly investigated. Moreover, endocrine therapy combined with cyclin-dependent kinase 4/6 (CDK4/6) inhibitors is now a standard of care in high-risk early breast cancer (13, 14). Preclinical data on palbociclib showed safety on ovarian function, but further evidence has to be provided (15).

PARP inhibitors are increasingly applied in the management of early breast cancer in germline BRCA-mutated patients (16), but their effects on fertility in humans remain unknown. Possible gonadotoxicity is suggested by preclinical studies in BRCA wild-type mice, which demonstrated that co-administration of olaparib with chemotherapy decreased primordial follicles by 36% compared to chemotherapy alone (p<0.05). Further preclinical and clinical studies are needed to assess reproductive safety.

Immune checkpoint inhibitors are increasingly used for triple-negative breast cancer in the neoadjuvant setting (17). Although evidence on their direct impact on POI is limited, these treatments can trigger inflammation of the thyroid, adrenal glands, or pituitary, potentially disrupting hormonal control of the hypothalamic-pituitary-ovarian axis and affecting fertility (18).

## 4 Subgroup considerations

- Hormone receptor–positive disease: Concerns about the oncologic safety of post-treatment pregnancy have been addressed by recent evidence. A meta-analysis by Lambertini et al. (19) found no increased risk of recurrence or mortality in women who conceived after breast cancer, including those with hormone receptor-positive breast cancer. Even better disease-free survival and overall-survival rates in BCY that subsequently had carried a pregnancy than those that did not; no higher risk of fetal abnormalities and reproductive complications was described (19). Although pregnancy requires temporary interruption of endocrine therapy, available data suggest no excess recurrence risk when conception occurs after at least 18–24 months of therapy (20).
- Triple-negative breast cancer: Data are more limited, but retrospective analyses indicate pregnancy does not worsen prognosis (19).
- HER2-positive disease: Evidence is scarce, especially in patients exposed to trastuzumab or novel HER2-targeted agents. Current evidence suggests delaying conception until completion of anti-HER2 therapy, at least 7 months for trastuzumab, pertuzumab, and TDM1 (19).
- BRCA mutation carriers: An international cohort study (21) also evaluated pregnancy rate and survival outcomes in BCY with germline BRCA mutations: in these patients, post-breast cancer pregnancy was safe, without compromise of maternal prognosis, and was associated with positive fetal outcomes. On the other hand, BRCA-mutated breast cancer patients consistently showed reduced reproductive potential and decreased success of cryopreservation strategies (22).

## 5 Timing and offspring outcomes

**I**dentifying the safest interval to pregnancy after treatment is influenced by both oncologic and obstetric considerations, since female cancer survivors have an elevated risk of adverse obstetric outcomes, including preterm delivery, low birth weight, increased rates of both elective and emergency caesarean section, greater need for assisted vaginal delivery, and higher incidence of postpartum hemorrhage (23).

The period of highest recurrence risk differs according to tumor subtype: in triple-negative disease, for instance, the first 2–3 years carry the greatest danger, which is why delaying conception until after this interval is often recommended. In women treated with adjuvant regimens, contraception must be maintained for several months following treatment completion, as pembrolizumab, atezolizumab, and olaparib are contraindicated during pregnancy (24, 25). For patients with HER2-positive tumors, childbearing requires additional planning. Trastuzumab and other anti-HER2 therapies have well-recognized fetal risks; therefore, effective contraception is mandatory during treatment and for up to seven months afterward (26). Similar caution applies to women receiving adjuvant endocrine therapy. Tamoxifen is teratogenic, and discontinuation is necessary before pregnancy attempts (27). While a three-month washout is generally recommended, shorter intervals may be pharmacologically acceptable, given the drug’s half-life of approximately seven days (20). Although long-term reproductive safety data are lacking, CDK4/6 inhibitors are contraindicated while attempting conception, and the monarchE trial required contraception for 12 weeks after the last dose (13).

## 6 Fertility preservation techniques

**Table 1** presents the advantages and disadvantages of FP techniques. Oocyte or embryo cryopreservation is considered the gold-standard FP approach for post-pubertal patients. GnRHa during chemotherapy is supported as an adjunct for ovarian function preservation but not a substitute for cryopreservation. Ovarian Tissue Cryopreservation (OTC) is recommended in urgent situations or when ovarian stimulation is not feasible.

**Table 1:** Fertility preservation strategies.

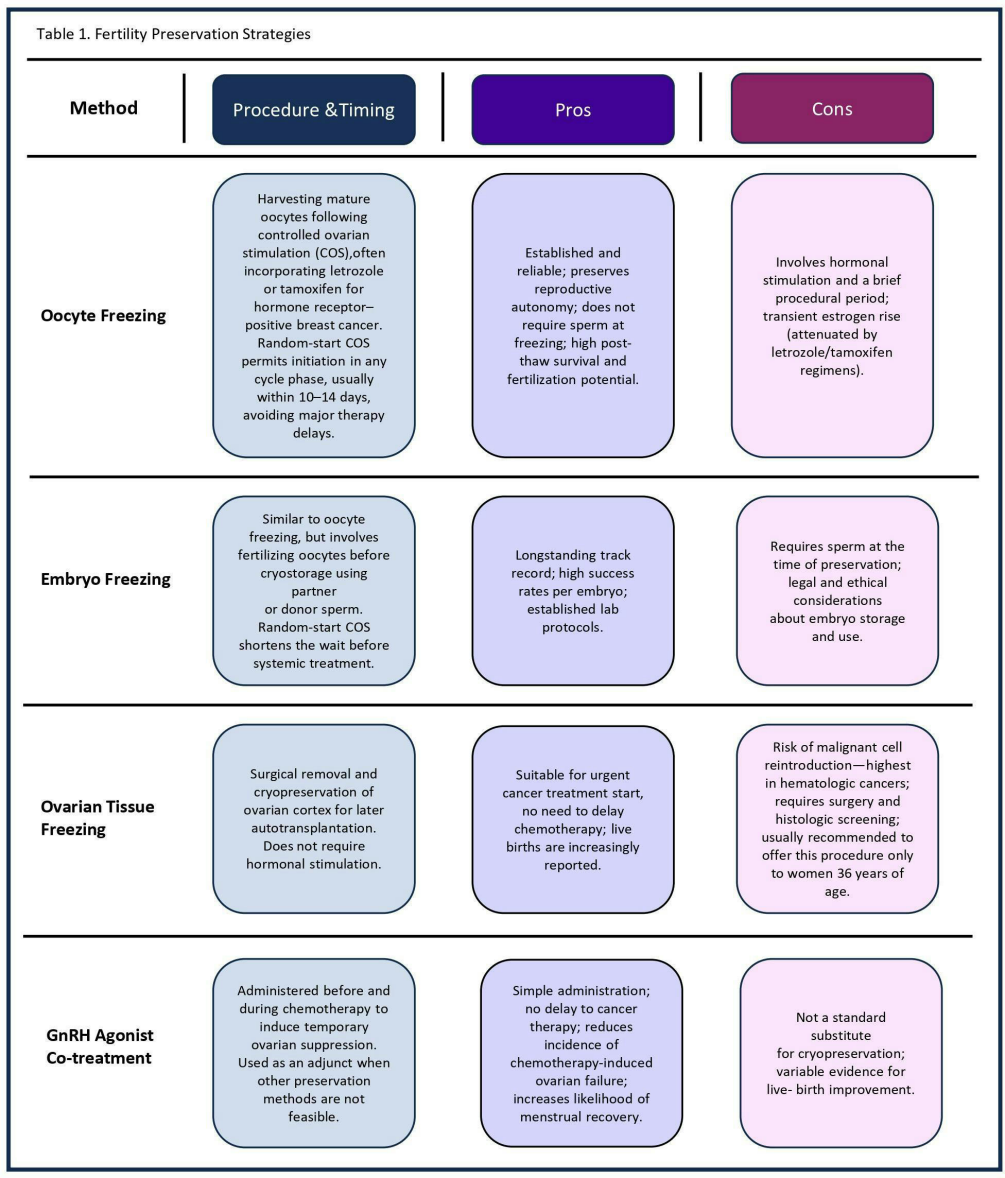

While consensus exists regarding the importance of early FP counseling, differences remain in the recommended hierarchy of methods (**Table 2**). The National Comprehensive Cancer Network (NCCN) recommends early discussion of FP, with oocyte or embryo cryopreservation as the standard, and OTC when treatment cannot be delayed (28, 29). ASCO similarly prioritizes oocyte and embryo cryopreservation, with weaker support for ovarian suppression alone (3). The Japan Society of Clinical Oncology (JSCO) also notes limited evidence for ovarian suppression (30). The Clinical Oncological Society of Australia (COSA) advises oocyte or embryo cryopreservation, with GnRH analogues to reduce chemotherapy-related ovarian failure (31). Canadian guidelines advocate for oocyte or embryo cryopreservation; GnRH agonists can be considered for gonadal cytoprotection before chemotherapy (32). Chinese guidelines suggest FP for patients aged <35 years with good ovarian reserve, emphasizing oocyte cryopreservation (33), while Korean guidelines caution that ovarian suppression should not substitute for established FP methods (34).

**Table 2:** Comparison of international guidelines on fertility preservation in breast cancer patients.

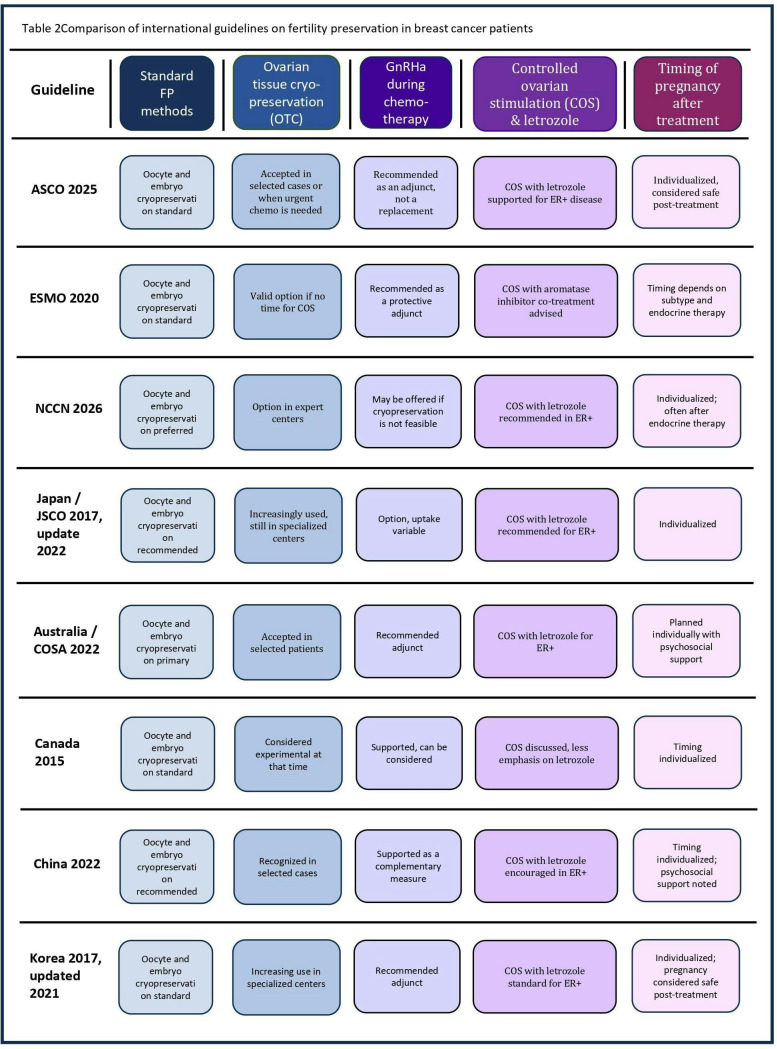

The main FP techniques will be analyzed in the following paragraphs.

### 6.1 Oocyte and embryo cryopreservation

Oocyte and embryo cryopreservation remain the most established FP methods, with high success rates and well-characterized safety profiles. Patients undergo approximately two weeks of ovarian stimulation with gonadotropins, culminating in follicle retrieval. Ovarian stimulation can be initiated at any point in the menstrual cycle (random-start protocols), minimizing treatment delays (35). Letrozole- or tamoxifen-supplemented stimulation protocols reduce peak estradiol levels, making the process safer for estrogen receptor-positive patients (36). Embryo cryopreservation is also an option, but it requires sperm from a partner or donor, whereas oocyte cryopreservation offers more autonomy and flexibility.

Despite robust outcomes, evidence about oocyte and embryo cryopreservation primarily comes from retrospective series and fertility clinic reports, which are subject to inherent selection bias (37). Although data on the long-term safety of ovarian stimulation in breast cancer survivors seem to be encouraging (38).

### 6.2 Ovarian tissue cryopreservation

OTC involves surgical removal of ovarian cortical tissue, which is cryopreserved for later autotransplantation. Among fertility preservation options, ovarian tissue cryopreservation is uniquely able to conserve both reproductive potential and endocrine activity (39). It is the only option for prepubertal patients and those requiring immediate chemotherapy (it has to be performed before gonadotoxic treatments). While concerns about reintroducing malignant cells persist, particularly in BRCA mutation carriers, advances in histological and molecular screening have improved safety (40). Evidence remains largely retrospective, with variable follow-up and potential reporting bias; nevertheless, an increased success rate in births has been reported worldwide following OTC (41, 42).

### 6.3 Gonadotropin-releasing hormone agonists

GnRHa administration during chemotherapy has been shown to reduce treatment-induced ovarian failure rates, avoiding premature ovarian insufficiency (POI) (43). A systematic review has demonstrated the safety of this method, ruling out any detrimental effect on survival in BCY (43). Adding GnRHa to chemotherapy often leads to a greater occurrence of menopausal complaints, particularly hot flushes and sweating. These symptoms are usually low-grade and tend to resolve once treatment is completed.

However, guidelines emphasize that GnRHa should not replace established cryopreservation techniques. Their use is most appropriate when other FP methods are not feasible due to time constraints or patient choice, or in conjunction with the other FP techniques. Indeed, evidence supporting GnRHa derives from two major randomized controlled trials, the POEMS and PROMISE-GIM6 studies (44–47). A meta-analysis of related trials supports these results (43). Nonetheless, most research to date has focused on chemotherapy-induced amenorrhea rather than direct measures of fertility; modest sample sizes, heterogeneous populations, and short follow-up also represent some limitations.

## 7 Interruption of endocrine therapy for pregnancy

Endocrine therapy, used for early endocrine-receptor positive tumors, consists of typically 5–10 years of tamoxifen with or without ovarian suppression and poses a challenge for women wishing to conceive soon after treatment. The POSITIVE trial (20) evaluated temporary interruption of endocrine therapy to attempt pregnancy. Eligible participants were women aged 42 years or younger with stage I–III hormone receptor–positive breast cancer who had completed between 18 and 30 months of adjuvant endocrine therapy and expressed the desire to pause treatment to pursue pregnancy. The 3-year rate of breast cancer events was 8.9% (95% CI, 6.3–11.6) among patients who paused treatment, compared with 9.2% (95% CI, 7.6–10.8) in the control arm. Limitations include: short follow-up, insufficient for late recurrences; highly selected patients (younger, lower-risk, motivated); a low number of recurrence events, limiting statistical power.

Until longer follow-up matures, clinicians should interpret findings with caution, recommending therapy interruption only for carefully selected patients, with a clear plan for resuming therapy postpartum. Such interruptions may be considered only after at least 18–24 months of therapy in patients with favorable disease characteristics, following thorough oncologic assessment (3, 4). After pregnancy and breastfeeding, therapy should be resumed to complete the planned duration.

## 8 Uptake, access, and psychosocial considerations

Despite strong recommendations, FP uptake remains suboptimal worldwide. Barriers include a lack of standardized referral processes, limited specialist availability, high out-of-pocket costs, and socioeconomic disparities. Cultural factors and patient perceptions also play a role, with some patients prioritizing oncologic treatment over FP or fearing that FP may compromise cancer outcomes (48).

Barriers from both physicians and patients can limit fertility preservation (FP) counseling in young women with breast cancer. Physicians who are knowledgeable and have positive attitudes are more likely to discuss fertility and provide referrals, yet systemic challenges, such as limited consultation time and poor collaboration with reproductive specialists, remain significant obstacles (49–52). Patients may hesitate to pursue FP due to cost, fear of cancer recurrence, or potential treatment delays (53, 54). Genetic considerations add complexity, as 5–10% of breast cancers are hereditary, most often involving BRCA1/2 mutations, which commonly occur during reproductive age and influence reproductive planning (55). Although evidence supports the safety and effectiveness of various FP strategies, data remain limited for BRCA mutation carriers, emphasizing the need for targeted research on fertility potential and post-diagnosis pregnancy (56). Educational tools, decision aids, and multidisciplinary programs improve patient knowledge, satisfaction, and referral rates, highlighting the importance of combining individualized counseling with systemic support to optimize FP care.

## 9 Future perspectives

Fertility preservation in young women with breast cancer is advancing rapidly, driven by progress in both reproductive medicine and oncology. AMH and antral follicle count (AFC) remain the most widely validated indicators of ovarian reserve, and combined use can improve the precision of individualized counseling (57). Ovarian tissue cryopreservation, once considered experimental, is now increasingly applied in clinical practice. Recent reports confirm its ability to restore endocrine function and achieve successful pregnancies following transplantation, with promising live-birth rates (58, 59).

Several ongoing trials are further shaping the field. A prospective study (NCT04586686) is evaluating whether combining ovarian biopsy with controlled ovarian stimulation can be performed safely without compromising oocyte yield. Another observational project (NCT04678414) aims to develop a comprehensive infertility survey for reproductive-age women with breast or gynecologic cancer to capture patient priorities better and guide counseling. In addition, the completed fAMHOPE study (NCT04289805) has evaluated the efficacy and safety of controlled ovarian stimulation, with or without letrozole, in newly diagnosed breast cancer patients candidated to receive (neo)adjuvant chemotherapy, with outcomes including mature oocyte yield, oncologic safety markers, and assisted reproduction results. Parallel advances are emerging from endocrine therapy interruption studies. Updated findings from the POSITIVE trial indicate that, in carefully selected patients, a temporary pause in adjuvant endocrine therapy to attempt pregnancy does not appear to worsen short-term cancer outcomes, while allowing favorable conception and live-birth rates (20).

Recent advances in FP include artificial-ovary constructs, *in-vitro* follicle growth (IVFG) platforms, decellularized extracellular matrix (dECM) scaffolds and microfluidic/organ-on-chip systems. dECM preparations have been shown to support follicle seeding; they are artificial ovaries, created by decellularizing the extracellular matrix and loading it with ovarian cells (60, 61). Despite the removal of most immunogenic material through decellularization, reports have documented persistent adverse outcomes, including inflammation, fibrotic tissue formation, and calcification. A central biological challenge is prompt and durable revascularization after implantation: ischemic loss due to delayed angiogenesis substantially reduces follicle survival and motivates integrated strategies combining pro-angiogenic factors, host-endothelial-cell co-seeding or pre-vascularized constructs. For IVFG, multistep culture systems and biomimetic scaffolds enable human follicle activation and growth to later stages *in vitro*; however, prolonged culture duration, species-scale differences, and the need to demonstrate the production of euploid, developmentally competent oocytes are substantial scientific and regulatory obstacles (62). Microfluidic technologies facilitate the precise manipulation of fluids within microscale channels to engineer organ-on-a-chip platforms that recapitulate key physiological and mechanical functions of human tissues and organs for applications in disease modeling and drug screening. They can better replicate physiological gradients and perfusion improving reproducibility and follicle/oocyte competence; device-to-device variability, choice of biomaterials, and integration with downstream clinical workflows must be systematically optimized and validated (63, 64). Finally, artificial-intelligence approaches show promise for protocol optimization, non-invasive quality assessment, and patient stratification, but models require large, multicenter training sets and prospective external validation to avoid bias and satisfy regulatory requirements (65, 66).

## 10 Discussion

Fertility preservation in young women with breast cancer has evolved from a peripheral consideration to a central component of comprehensive oncologic care. Contemporary guidelines from ASCO and ESMO converge on the principle that all women of reproductive age should be offered timely counseling on potential treatment-related gonadotoxicity and fertility preservation strategies before the initiation of systemic therapy (3, 4). This approach is supported by increasing survival rates in young breast cancer patients and the recognition that quality-of-life outcomes, including the ability to conceive, significantly influence survivorship satisfaction.

Despite the availability of evidence-based recommendations, real-world uptake of fertility preservation remains suboptimal. Barriers include delayed or absent referral to reproductive specialists, variability in clinicians’ awareness of guideline recommendations, and logistical constraints related to the urgency of systemic therapy initiation. Literature underscores the necessity of embedding reproductive counseling within the multidisciplinary tumor board process, ensuring early referral to reproductive endocrinology without compromising oncologic timelines.

The choice of fertility preservation strategy is guided by the patient’s age, ovarian reserve, tumor biology, and treatment urgency. Oocyte and embryo cryopreservation remain the gold-standard options, with live birth rates in oncologic patients now approaching those in non-cancer populations when undertaken in specialized centers (67). OTC is gaining traction, particularly for patients requiring immediate systemic therapy or those prepubertal at diagnosis. While OTC is still considered experimental in some jurisdictions, accumulating safety and efficacy data — including reports of restored endocrine function and live births — support its cautious inclusion in selected patients.

Pharmacologic ovarian suppression with GnRHa during chemotherapy has been shown in multiple randomized controlled trials and meta-analyses to reduce the risk of premature ovarian insufficiency (43). However, its role as a stand-alone fertility preservation method remains controversial. Current evidence and guidelines position GnRHa as an adjunct to, rather than a replacement for, cryopreservation techniques.

Post-treatment reproductive planning is an area of increasing clinical relevance. Historically, clinicians advised delaying pregnancy for several years after breast cancer diagnosis, primarily out of concern for recurrence risk. Large-scale meta-analyses and pooled cohort studies have consistently shown that pregnancy after breast cancer does not worsen disease-free or overall survival, even in hormone receptor–positive subgroups. The POSITIVE trial provides prospective evidence that temporary interruption of adjuvant endocrine therapy to attempt conception is feasible and does not appear to confer an excess short-term recurrence risk (20). The trial emphasizes the importance of careful patient selection, oncologic reassessment, and a structured plan for resuming therapy after pregnancy and lactation.

BRCA mutation carriers warrant specific consideration due to their increased baseline risk for both breast and ovarian cancers. Fertility preservation discussions in this group must address the potential timing of risk-reducing salpingo-oophorectomy and the possibility of preimplantation genetic testing to prevent transmission of pathogenic variants. Coordination between oncologists, reproductive endocrinologists, and genetic counselors is critical.

## 11 Conclusions

Fertility preservation is a central survivorship consideration for young women with early breast cancer. Oocyte and embryo cryopreservation should be offered as first-line options, with OTC and GnRHa as alternatives when standard methods are not feasible. Current evidence supports the safety of pregnancy after breast cancer, including hormone receptor-positive and BRCA mutation carriers.

Endocrine therapy interruption may be considered for carefully selected patients wishing to conceive, guided by ongoing trial data and multidisciplinary evaluation. Closing the gap between evidence and practice will require improved access to FP services, early and consistent counseling, and coordinated care pathways that respect each patient’s reproductive goals within the context of optimal oncologic management.
